# Sporadic Desmoid Tumor Mimicking Myofascial Pain Syndrome in a Chiropractic Clinic

**DOI:** 10.7759/cureus.44653

**Published:** 2023-09-04

**Authors:** Eric Chun-Pu Chu, Edouard Sabourdy

**Affiliations:** 1 Chiropractic and Physiotherapy Centre, New York Medical Group, Hong Kong, CHN; 2 Chiropractic Clinic, Franco-Vietnamese (FV) Hospital, Ho Chi Minh City, VNM

**Keywords:** basic fibroblast growth factor, chiropractor, chiropractic, fibroblast, desmoid tumor

## Abstract

Chiropractors are primary healthcare providers who diagnose and manage various health conditions, including rare cases like desmoid tumors. Desmoid tumors are locally aggressive soft-tissue tumors originating from fibroblasts. This report presents the case of a 30-year-old woman who initially sought chiropractic care for persistent neck pain that was later discovered to be a symptom of a sporadic desmoid tumor. The patient presented with severe right upper neck pain, which was diagnosed as myofascial pain syndrome and yielded no significant improvement after various treatments. Physical examination by a chiropractor revealed a soft-tissue mass in the right upper trapezius and rhomboid region. In addition, magnetic resonance imaging revealed an intramuscular lesion measuring 8 × 4 cm in the right rhomboids, and subsequent biopsy confirmed the diagnosis of a sporadic desmoid tumor. The patient underwent successful surgical excision of the tumor. Postoperatively, the chiropractor initiated a comprehensive 12-week rehabilitation program that significantly improved the patient's range of motion and muscular strength and alleviated pain. The remarkable aspect of this case was the location of the tumor in the right rhomboid muscle, a less common site for desmoid tumors. This case underscores the crucial role of chiropractors as primary healthcare providers in the early detection of oncological cases and management of post-surgical rehabilitation.

## Introduction

As primary healthcare providers, chiropractors play a significant role in diagnosing and managing various health conditions [1]. Their comprehensive approach to patient care extends beyond musculoskeletal disorders and encompasses a wide range of conditions, including oncological cases [2-7]. Chiropractors utilize a unique set of skills and techniques, combining detailed physical examinations with clinical acumen to detect abnormalities that may otherwise go unnoticed [8]. This case report presents a distinctive case where a chiropractor played a crucial role in the initial detection of a sporadic desmoid tumor in the right rhomboid muscle -- an unusual location that adds to the rarity of this case. 

Desmoid tumors are rare, soft-tissue tumors that originate from fibroblasts and exhibit a locally aggressive behavior [9]. Although benign, these tumors are known for their infiltrative growth pattern, which enables them to invade adjacent anatomical structures, entrap them, and cause substantial degeneration [9]. The diagnosis of desmoid tumors is challenging because of their diverse morphological features and variable clinical manifestations at an atypical location of the tumor [9]. Patients frequently consult multiple healthcare providers before receiving a correct diagnosis and often face delays in diagnosis during their clinical course [9].

This case report presents a novel example of a chiropractor playing a crucial role in the initial detection and subsequent management of a sporadic desmoid tumor in a patient presenting with severe, persistent neck pain. This report highlights the significant role played by chiropractors in the early detection of oncological conditions and their critical involvement in the management and post-surgical rehabilitation of such patients.

## Case presentation

A 30-year-old woman presented to the chiropractic clinic with a chief complaint of right upper neck pain rated 8/10 on a numeric pain scale. She described the pain as a spasm in the right upper trapezius that radiated to her upper neck, midback, suboccipital region, and collar bone on the same side. The patient had been experiencing this pain for the past three years, with a strong exacerbation for a week, leading to decreased cervical range of motion, particularly affecting neck extension. The pain interfered with her daily activities and sleep. There was no significant family history or genetic predisposition to oncological conditions. The patient's psychosocial history was unremarkable. Her quality of life was rated as 72% based on the World Health Organization Quality of Life score (WHOQOL-BREF).

**Figure 1:**
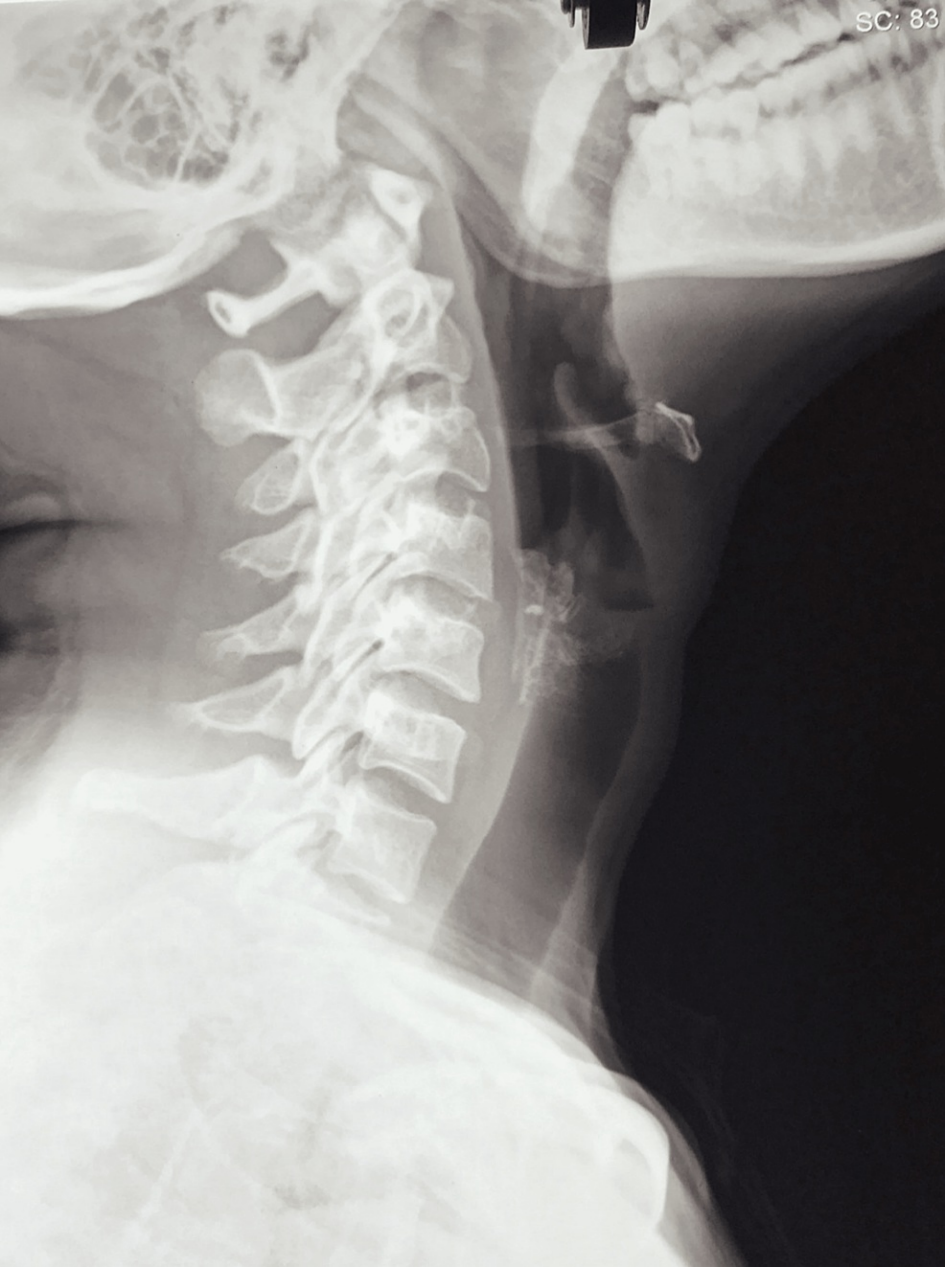
Cervical radiograph showing no abnormalities

Before her visit to the chiropractic clinic, an orthopedic surgeon performed cervical radiography, which showed no abnormalities (Figure 1). The patient was subsequently diagnosed with myofascial pain syndrome and treated with 12 sessions of rehabilitation exercises, 20 sessions of massage therapy, and 24 sessions of acupuncture. Despite these treatments, her pain persisted, and she was referred to a chiropractor.

On physical examination, the chiropractor did not observe any visible abnormalities or asymmetries in the cervical and thoracic regions. The range of motion was restricted to the cervical and thoracic regions, particularly during extension, right lateral flexion, and rotation. Right arm abduction was rated 4/5 without any other motor or sensory deficits or signs of muscle atrophy or weakness. However, a soft-tissue mass with localized tenderness and mild swelling was detected in the right upper trapezius and rhomboid regions.

**Figure 2:**
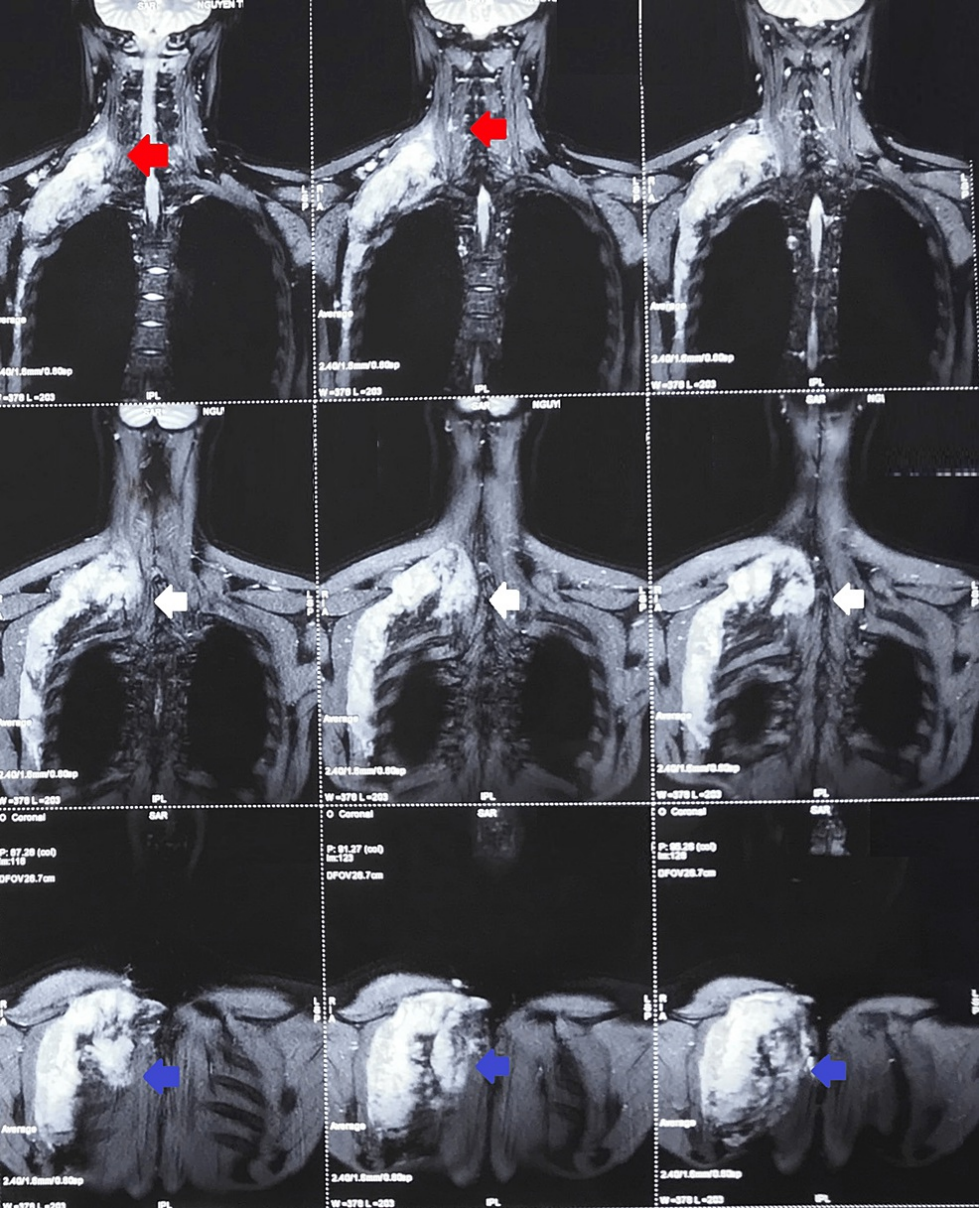
Magnetic resonance imaging (MRI) scans conducted on the upper back region There is a notable mass observed within the rhomboid muscles (write arrows), encompassing both the minor and major parts. This lesion demonstrates hyperintensity on T2-Weighted (T2W) images. In post-contrast images, the mass reveals a homogeneous strong enhancement. The lesion exhibits invasive characteristics, with evidence of infiltration into the longissimus cervicis muscle (red arrows) and the infraspinatus muscle (blue arrows). The MRI features are suggestive of a desmoid tumor in the rhomboid muscles with invasion into the longissimus cervicis and infraspinatus muscles.

Due to persistent pain and the detected mass, the chiropractor prescribed a magnetic resonance imaging (MRI) scan for the upper back region, which revealed the presence of an intramuscular lesion of 8 × 4 cm. This lesion was found to penetrate the cervical area of the right rhomboid muscle, without any involvement of the superficial subcutaneous tissues or the skin (Figure 2). Detection of the mass posed a diagnostic challenge as the initial symptoms were suggestive of myofascial pain syndrome, emphasizing the importance of a thorough physical examination.

An incisional biopsy was performed by an oncologist in the same clinic, who established the diagnosis of a sporadic desmoid tumor. The prognostic characteristics of desmoid tumors depend on their size, location, and growth rate. Although benign, they can cause significant morbidity owing to local invasion and potential recurrence.

The chiropractor and the affiliated surgeon engaged in a discussion over the potential option of adopting a vigilant approach to monitoring the patient's condition before contemplating surgical intervention. Nevertheless, the patient made the decision to have surgical intervention, a choice that was deemed rational in light of the symptoms she was presenting. The oncologist and associated surgeon successfully excised the sporadic desmoid tumor in the right rhomboid region, ensuring complete removal with clear margins to minimize the risk of recurrence.

Postoperatively, the patient was referred back to the chiropractor to initiate a comprehensive 12-week rehabilitation program. The program aimed to restore her full range of motion, improve muscular strength and endurance, and alleviate any residual pain or discomfort. Throughout the postoperative rehabilitation process, the patient attended regular follow-up appointments with the chiropractor to monitor her progress and adjust her exercise program, as needed.

At the end of the 12-week rehabilitation program, the patient reported significant improvements in her range of motion, muscular strength, and overall function. The chiropractor also utilized manual therapy techniques, such as joint mobilization, instrument-assisted soft-tissue mobilization, and soft-tissue release, to complement the exercise program and further facilitate patient recovery. Her pain score decreased from 8/10 to 1/10 on the numeric pain scale. Her quality of life rating improved to 100%, and she adhered well to the therapeutic interventions and reported no adverse or unanticipated events. The patient was advised to continue a maintenance exercise program and follow-up with her chiropractor and oncologist, as needed, to monitor her long-term recovery and ensure the absence of tumor recurrence.

## Discussion

Desmoid tumors are rare benign tumors with an incidence of three to five cases per million people per year [9]. These tumors often impose a high symptom burden; approximately 63% of the patients experience chronic pain, leading to sleep disturbances, irritability, and sometimes anxiety or depression [9]. Symptoms commonly include pain, limited mobility, fatigue, muscle weakness, and swelling around the tumor, causing a decrease in the overall quality of life compared with that of healthy individuals [9].

In this case report, we highlight the pivotal role that a chiropractor can play in the early detection of rare oncological conditions, such as desmoid tumors, and in patient management during postoperative rehabilitation. The patient initially sought chiropractic treatment for persistent neck pain, a common complaint in the field. However, the chiropractor’s keen clinical judgment uncovered an uncommon pathology. This emphasizes the importance of thorough physical examinations by chiropractors as primary healthcare providers and their ability to manage a wide spectrum of health conditions, including rare oncological cases.

A remarkable aspect of this case was the chiropractor’s insightful recommendation of an MRI, despite the patient's prior diagnosis of myofascial pain syndrome. This critical step led to the detection of the desmoid tumor, highlighting the significance of maintaining a high level of suspicion when dealing with persistent or unusual symptoms. However, the rarity and atypical presentation of desmoid tumors present a diagnostic challenge, possibly leading to delayed detection and management. The uniqueness of the patient's presentation and rarity of the condition may limit the generalizability of this case.

Although no specific treatment for desmoid tumors has been approved, current guidelines suggest several options, such as active surveillance, surgery, systemic therapy, and locoregional therapy [9]. Treatment selection is typically based on tumor location, symptoms, and potential morbidity [9]. Notwithstanding these options, there remains a significant need for more targeted treatments that can effectively manage desmoid tumors and enhance the patients' quality of life [9].

This case report underscores the vital role of chiropractors in the early detection of oncological conditions and their significant contribution to managing and rehabilitating patients postoperatively. This serves as a reminder to chiropractors to be vigilant when faced with persistent and nonresponsive symptoms.

## Conclusions

This case report illuminates the multifaceted role of chiropractors as a part of an interdisciplinary healthcare team. It underscores how vigilant assessment and clinical judgement can lead to the detection of rare and unusual pathologies, such as desmoid tumors. The case also emphasizes the importance of maintaining a high level of suspicion for atypical presentations, especially in patients with persistent, non-responsive symptoms. Furthermore, the successful postoperative rehabilitation managed by the chiropractor demonstrates the significance of comprehensive, holistic patient care. This case serves as a reminder of the potential impact chiropractic care can have on patient health outcomes and reinforces the need for ongoing research into rare conditions, such as desmoid tumors, to improve their diagnosis and treatment.
